# Social and health reasons for lime juice vaginal douching among female sex workers in Borno State, Nigeria

**DOI:** 10.4102/phcfm.v2i1.125

**Published:** 2010-05-21

**Authors:** Abdulkarim G. Mairiga, Abubakar A. Kullima, Mohammed B. Kawuwa

**Affiliations:** 1 Department of Obstetrics and Gynaecology, University of Maiduguri, Nigeria

**Keywords:** female sex workers, lime juice, Nigeria, social reasons, vaginal douching

## Abstract

**Background:**

Vaginal douching with lime juice and other agents has been perceived to enhance sexual excitement through sensations of vaginal dryness, tightness or warmth, as well as prevent sexually transmitted infections and restore and tighten the vagina after delivery. Its effectiveness as a contraceptive has also been reported. However, the social and health reasons/consequences of such a practice have not been adequately documented in the communities of Borno State, Nigeria.

**Objectives:**

This study aimed to determine the extent of, reasons for, and the reproductive health effects of, the use of lime juice for vaginal douching among the commercial sex workers (CSWs) in selected areas of the three senatorial regions of Borno State, Nigeria.

**Method:**

This was a community-based descriptive study conducted among female CSWs in selected communities of Borno State, Nigeria. A sample of 194 CSWs were randomly selected and interviewed on their sexual history and douching practices.

**Results:**

One hundred and twenty (62%) respondents admitted practicing vaginal douching with lime juice, with 85% having been CSWs for a period greater than three years. More than half douched for sexual pleasure, hygiene and contraception. Significantly more lime juice users had increased susceptibility to sexually transmitted infections (STIs) than non-users. Users had a higher prevalence of HIV infection than non-users. The Papanicouleaua (pap) smear test for cervical lesions also showed that moderate to severe dysplastic changes were more prevalent among lime-juice users.

**Conclusion:**

Many CSWs in this community use lime juice for douching, for various reasons. Indications are that its use is associated with a higher prevalence of sexually transmitted diseases, including HIV infections and dysplastic cervical changes. Owing to confounding issues, such as the number of sexual partners, frequency of sexual exposure per day and the duration of exposure, it cannot, therefore, be deduced that douching with lime juice is the only reason for the higher prevalence of STIs and HIV. Nevertheless, there is an obvious need to mount extensive campaigns to educate the CSW on the possible risks of using such a practice.

## INTRODUCTION

Vaginal douching is a global traditional practice dating back hundreds of years, with the perception that it enhances sexual pleasure.^1^ It is a particularly common practice among both married women (who are not sex workers) and female sex workers in many parts of sub-Saharan Africa.^2^ Vaginal douching with lime juice has been perceived among women to enhance sexual excitement through sensations of vaginal dryness, tightness or warmth. Vaginal cleansing before or after, or between, the act of intercourse is thought to treat gynaecological diseases, prevent sexually transmitted infections and restore and tighten the vagina after delivery.^1–4^ Its contraceptive effectiveness has also been reported.^5^

Increasingly, evidence also suggests some adverse reproductive health effects of vaginal douching agents (including lime juice), which includes increased risks of bacterial vaginosis, HIV infection, pelvic inflammatory disease (PID) and cervical cancer.^6–8^

Anecdotal information from health workers within Borno State, Nigeria reports that a significant number of female commercial sex workers (CSWs) in the area douched with lime juice before or after sexual intercourse.^9^ This study was therefore undertaken to determine the extent of, reasons for, and the reproductive health effects of, lime juice use for vaginal douching among the CSWs in selected areas of the three senatorial regions of Borno State, Nigeria.

## METHOD

### Study area

Borno State is one of the 36 states of the Federal Republic of Nigeria and is located in the north-eastern corner of the country. The state occupies a greater part of the Chad Basin area borders three African countries, namely, Niger to the north, Chad to the north-east and Cameroon to the east. Within Nigeria, it shares borders with Yobe, Adamawa and Gombe States to the north-west and south, respectively. There are 27 local government areas (LGAs) in the state and Maiduguri is the state capital.

The state covers an area of 69 436 km^2^ and has a population of 4 151 193 (according to the 2006 national population census), with 64.37% of the populace living in rural areas and 35.63% in urban.^10^ Islam is the major religion, followed by Christianity. The principal tribes of the state are the Kanuri, followed by the Babur, Bura, Marghi, Shuwa Arabs, Fulani and the Hausa. The state is rich with various cultures, norms and values, with the popular occupations of Borno citizenry including farming, fishing and trading.

Available statistics show that the reproductive health situations in this area are the worst, compared to that of any other part of Nigeria. The crude birth rate is 43.60 per 1000, the gross fertility rate is 183 per 1000 and the maternal mortality ratio is 1549 per 100 000 live births. Only 2% of women in this region are said to be using modern contraceptives.^11^ The HIV/AIDS prevalence has been fluctuating from 4.5% to 5.4%; the current 2003 sentinel survey amongst women attending ante-natal clinics indicated the prevalence at 3.2%. However, this prevalence rate of 3.2% is not realistic as hot-spot areas, such as border towns^12^ (e.g. Baga, Ngala and Askira/Uba), were not included in the survey.

This study was conducted in three LGAs: the Metropolitan Council of Maiduguri, Ngala and Askira/Uba, representing the three senatorial zones of Borno State, Nigeria. Maiduguri Metropolitan Council is the state capital, while Ngala and Askira/Uba are two border towns with significant numbers of CSWs.^12^ This study was conducted between 04 and 30 July 2008.

### Study participants

A sample of 194 CSWs were randomly selected and interviewed, both in formal (brothel) and informal settlements in the three LGAs. Simple random sampling was used to select brothels in each of the communities studied, with the leader of the CSWs in each brothel also randomly selecting the CSWs for the interview. Exclusion criteria were the use of barrier and hormonal contraceptives and the use of other douching methods, such as medicated creams, lotions or soaps. Finally, the CSWs were taken to a clinic for pelvic examination, endocervical and high vaginal swabs, the Papanicouleaua (pap) smear and venous blood sampling for HIV screening. Information pertaining to their demographic data, sexual history (duration of sexual exposure, number of sexual partners and frequency of sexual exposure over a period of time) and history of sexually transmitted infections (STIs) were obtained. Further information on the douching practice with lime juice was also obtained, which included the duration of this practice, the frequency of douching relative to sexual exposure, when douching was carried out in relation to sexual activity, concentration of the lime juice used, and reason/s for the douching (e.g. health, social, contraception). This information was obtained on a one-to-one basis, confidentiality was assured to the CSWs and informed consent obtained. Each of the CSWs visited the clinic during clinic hours and was seen as if she had come for a routine consultation. Each of the CSWs came to the clinic with a referral note which was given to the consulting doctor only.

Pelvic examination and specimens of the endocervix and high vaginal swabs were collected for microbiological evaluation of *Neisseria gonorrhoea, Candida albicans, Trichomonas vaginalis* and Bacterial vaginosis. A pap smear was also obtained to evaluate for possible cervical lesions. After voluntary confidential counselling, specimens of venous blood were obtained for serologic tests for HIV.

Data were analysed using the statistical package SPSS version 11.0 and the chi-square test was used for test of significance using Epi info 2002 version. A *p*-value of 0.05 was considered significant.

## RESULTS

Of the 194 CSWs interviewed, 120 (62%) practiced vaginal douching with lime juice and the remaining 74 (38%) did not douche. One hundred and three (85%) users had been CSWs for more than 3 years and only 39 (50%) of non-users had been CSWs for longer than 3 years (see Table 1).

**TABLE 1:** Sexual history of respondents (*N* = 194)

	Users (*n* = 120)	%	Non-users (*n* = 74)	%
Duration of sexual exposure*				
< 3 years	17	14.2	35	47.3
3–6 years	63	52.5	19	25.7
7–10 years	40	33.3	20	27.0
**Number of sexual partners per day**†				
3–5	68	56.7	54	73.0
6–10	38	31.7	18	24.3
> 10	14	11.6	2	2.7
**Frequency of sexual exposure per day**‡				
1	50	41.7	32	43.2
2–5	42	35.0	28	37.8
6–10	28	23.3	14	19.0

* significant (*p =* 0.00; *df =* 2; χ^2^ = 27.13)

† significant (*p* = 0.03; *df* = 2; χ^2^ = 7.25)

‡ not significant (*p* = 0.06; *df* = 2; χ^2^ = -0.78).

More non-users had fewer sexual partners (< 5 per day) than lime-juice users: 54 (73%) compared to 68 (56.7%); however, significantly more lime-juice users (11.6%) had more sexual partners (> 5 per day) than non-users (2.7%). Overall, there was no significant difference between users and non-users with respect to the frequency of sexual exposure per day (see Table 1). The various perceived reasons for the douching practice and douching frequency patterns are given in Table 2.

**TABLE 2:** Reasons for and frequency of vaginal douching with lime juice

	Users (*n* = 120)	%
**Reasons**		
Hygiene*		
Sexual pleasure*	80	66.7
Infection prevention*	50	42.0
Contraception*	60	50.0
**Frequency of douching practice**		
Daily	38	31.7
With every act of coitus	70	58.3
Before and after sexual exposure	12	10.0

* Multiple responses.

This study showed that 66.7% of users douched for sexual pleasure and hygiene to reduce vaginal odour with or without sexual exposure, while 50% and 42% of the users douched for contraceptive reasons and infection prevention, respectively.

Seventy users (58.3%) douched with lime juice with every act of sexual exposure, compared to 38 (31.7%) that douched daily (with or without an act of sexual exposure). More users (54, 45%) applied a mixture of one part lime juice to four parts water, compared to those (39, 32.5%) who used a 1:1 mixture of lime juice and water.

Table 3 shows the results of a microbiological analysis and a serological test for HIV and other STIs. Multiple organisms were isolated in both the users and the non-users, but significant numbers of users harboured more *Neisseria gonorrhoea* (56.7%), *Trichomonas vaginalis* (65%) and Bacterial vaginosis (58.3%) than non-users, with 40.5%, 43% and 27% of the organisms, respectively. The serological test for HIV was higher in users, 58 (48.3%), compared to non-users, 29 (39.2%). The pap smear results tabulated in Table 4 show that there were more cases of moderate to severe dysplastic cervical changes among users (35; 29.2%) than non-users (10; 13.5%), but there were more inflammatory cells and mild dysplastic changes among non-users (24; 32%) than users (27; 22.5%).

**TABLE 3:** The correlation between sexually transmitted infections and the use of lime juice

Sexually transmitted infection	Users (*n* = 120)	%	Non-users (*n* = 74)	%
Neisseria gonorrhoea*	68	56.7	30	40.5
Trichomonas vaginalis*	78	65.0	32	43.0
Bacterial vaginosis*	70	58.3	20	27.0
Candida albicans*	30	25.0	15	20.0
HIV*	58	48.3	29	39.2

Not significant (*p* = 0.51; *df* = 4; χ^2^ = 3.30)

* Muliple organisims isolated.

**TABLE 4:** Pap smear results of lime-juice users and non-users

Smear grades	Users (*n* = 120)	%	Non-users (*n* = 74)	%
Normal cells	58	48.3	40	54.0
Inflammatory cells and mild dyskaryosis	27	22.5	24	32.0
Moderate and severe dyskaryosis	35	29.2	10	13.5

Significant (*p* = 0.03, *df* = 2, χ^2^ = 6.85).

## DISCUSSION

The main objective of this study was to determine the social and health reasons for vaginal douching with lime juice among CSWs in Borno State, Nigeria. The study shows that douching practices are common among CSWs in the community and the reasons include sexual pleasure, hygiene and contraception. Significantly more lime-juice users have increased susceptibility to STIs than non-users. More of the users have a higher prevalence of HIV infection than non-users. The pap smear test for cervical lesions also show that moderate to severe dysplastic changes are more prevalent among lime-juice users.

Although this is a descriptive study that lacks a control of variables, it has the advantages of being community-based and it provides the baseline data of douching practices among the CSWs from some communities in Nigeria, as well as the influence of this practice on the prevalence of STIs, including HIV and uterine cervical lesions. However, it must be noted that confounding factors, such as the number of sexual partners, period of time spent in commercial sex work and the number of sexual encounters per day, do not allow us to conclude that douching practice is the only act responsible for the high prevalence of STIs in the CSWs.

As a result of this study it was found that vaginal douching is common among CSWs, with a prevalence of 62%, which correlates with an earlier report of 72% among female sex workers in Nairobi, Kenya^1^, a similar African setting. However, our figure (62%) is much higher than that reported in the USA (37%) among females of reproductive age.^13^ It is possible that non-CSWs that constituted the population in the latter study made the prevalence much lower.

Our findings also revealed that a significant number of lime-juice users have been practicing CSWs for a longer period (> 3 years) and also had more sexual partners, when compared to non-users. This might partly explain the influence of the period of sexual practice and number of sexual partners on the douching practice, which also correlates with findings in central Africa.^7^ In the sexual history of the respondents, there were significant differences between the duration of sexual exposure and the number of sexual partners between the lime-juice users and non-users, while the frequency of daily sexual exposure was not a significant differential factor between the groups.

The majority of lime-juice users in this study (66.7%) douched for sexual pleasure and hygiene – a common reason reported for douching, worldwide^3, 13–15^ – while about 50% douched for contraceptive reasons as well (this also collaborated with US reports).^3, 5^ Only less than half assumed that they douched to prevent infection, as was similar to the assumption among patients enrolled for gamete intra-follicular transfer practicing vaginal douching.^3^

More of the lime-juice users were observed to harbour *Neisseria gonorrhoea* (56.7%), *Trichomonas vaginalis* (65%) and Bacterial vaginosis (58.3%) than non-users (40.5%, 43% and 27%, respectively). This was similar to results of earlier studies correlating an increased risk of these infections and vaginal douching,^1–4, 16, 17^ while the HIV seroprevalence was equally more among lime-juice users (25%) than non-users (20%). This seems to be compounded by the higher number of sexual partners and the duration of sexual exposure among lime-juice users, hence this relationship might be defined by multiple factors, rather than being attributed to the use of lime juice alone, which might have played an indirect role. Similar relationships among the above factors have been reported earlier in both developed and developing countries.^1, 18^

There was also a greater likelihood of developing cervical cancers among lime-juice users than non-users, as suggested by the findings of a higher prevalence of moderate to severe dysplastic changes among users (29.2%) than non-users (13.5%). This was statistically significant and a similar correlation has been reported by others.^1, 19^ Similarly, this finding might also be attributed to multiple factors, such as the number of sexual partners and duration of sexual exposure, and possibly the douching pattern and concentration of lime juice used.

## CONCLUSION

In conclusion, many CSWs in the communities of Borno State, Nigeria use lime juice for douching for various reasons, including hygiene, sexual pleasure, contraception and infection prevention. The use of lime juice has been associated with a higher prevalence of STIs, including HIV infections and dysplastic cervical changes. Because there are confounding issues, such as the number of partners, frequency of exposure per day and the duration of exposures, it can, therefore, not be deduced that douching is the only reason for the higher prevalence of STIs and HIV. Nevertheless, there is an obvious need to launch extensive campaigns to educate the CSW on the possible risks of using such a practice.
